# Fabrication and Characterization of Black GaAs Nanoarrays via ICP Etching

**DOI:** 10.1186/s11671-021-03479-1

**Published:** 2021-01-21

**Authors:** Jing Ma, Yongqiang Zhao, Wen Liu, Peishuai Song, Liangliang Yang, Jiangtao Wei, Fuhua Yang, Xiaodong Wang

**Affiliations:** 1 Engineering Research Center for Semiconductor Integrated Technology, Institute of Semiconductors, Chinese Academy of Science, Beijing, 100083 China; 2 Jihua Laboratory, Foshan, 528200 China; 3 Center of Materials Science and Optoelectronics Engineering, University of Chinese Academy of Sciences, Beijing, 101408 China; 4 School of Microelectronics, University of Chinese Academy of Sciences, Beijing, 101408 China; 5 Beijing Academy of Quantum Information Science, Beijing, 100193 China; 6 Beijing Engineering Research Center of Semiconductor Micro-Nano Integrated Technology, Beijing, 100083 China; 7 State Key Laboratory for Superlattices and Microstructures, Institute of Semiconductors, Chinese Academy of Sciences, Beijing, 100083 China

**Keywords:** Nanostructures, Black GaAs, ICP etching, Hydrophobic

## Abstract

GaAs nanostructures have attracted more and more attention due to its excellent properties such as increasing photon absorption. The fabrication process on GaAs substrate was rarely reported, and most of the preparation processes are complex. Here, we report a black GaAs fabrication process using a simple inductively coupled plasma etching process, with no extra lithography process. The fabricated sample has a low reflectance value, close to zero. Besides, the black GaAs also displayed hydrophobic property, with a water contact angle of 125°. This kind of black GaAs etching process could be added to the fabrication workflow of photodetectors and solar cell devices to further improve their characteristics.

## Introduction

Owing to its unique optical properties, light-trapping structure plays a more and more important role in photovoltaic devices [1]. At present, researchers have developed all kinds of nanostructures as light-trapping structures to increase light absorption in photovoltaics, while most of them were performed on Si substrate [2–6]. III–V compound semiconductor nanostructures have been shown to be promising materials for a variety of optoelectronic and energy-related applications such as light-emitting diodes (LEDs) [7, 8], photovoltaics (PV) [9–12] and field effect transistors (FETs) [13–16]. GaAs is a promising candidate as its direct bandgap and absorption property [17, 18]. When incident light enters the nanostructure, the photons will undergo multiple reflections and refract inside the structure and get trapped in the array, which is the trapping effect of nanostructure. And because of the absorption characteristics of GaAs materials, it means that more photon energy is absorbed by GaAs [19, 20]. However, compared with Si nanoarray structure, the research on GaAs nanoarray structure is relatively reported.

For the preparation process of GaAs nanoarrays, researchers from the University of Illinois [21] presented a GaAs nanopillar array with soft lithography and metal-assisted chemical etching (MacEtch) process in the year of 2011. The fabricated nanostructures have uniform width which can be used in optoelectronic devices and optical detectors. The researchers from Chinese Academy of Science [19] analyzed the properties of GaAs nanoarray anti-reflection resistance through theoretical simulation with finite-difference time-domain (FDTD) software, providing a detailed theoretical reference for the optical properties of nanostructures. In 2012, Lee et al. [22] prepared sub-micron nanoarray structures on GaAs substrate using colloidal crystal lithography barrier layer, which had been widely used in solar cells. In 2016, Song et al. [23] fabricated GaAs subwavelength structures by Au-assisted chemical etching. The fabricated GaAs structures dramatically reduced the total reflectance to 4.5% in a wavelength range of 200—850 nm up to the incident angle of 50°. In 2018, Paola Lova et al. [24] demonstrated anisotropic metal-assisted chemical etching of GaAs wafers exploiting the lower etching rate of the monoatomic Ga ˂111˃ and ˂311˃ planes. They also proposed a qualitative reaction mechanism for anisotropic etching of GaAs and showed that reflectance of the roughened surface of black GaAs reduces up to ~ 50 times compared to polished wafers. In 2020, Paola Lova et al. [25] proved that the etched GaAs (black GaAs) presented satisfactory light-trapping properties and the etched sample attracted more photon recycling. The articles mentioned above all proved that GaAs nanometer array structure has excellent photoelectric properties. But most of them are fabricated through metal-assisted etching, which requires complicated chemical process and the disposal of waste liquid such as HF is also troublesome. Moreover, Au is used as the auxiliary metal, and the cost is relatively high.

So here we demonstrate a black GaAs fabrication process using a simple inductively coupled plasma (ICP) etching process, and no extra lithography process, etc. The fabricated sample has a low reflectance value, close to zero. Besides, the black GaAs also display hydrophobic property, with a water contact angle (CA) of 125°. On the whole, this kind of black GaAs etching process could be added to the fabrication workflow of photodetectors and solar cell devices to further improve their characteristics.

## Methods

### Black GaAs Nanoarrays Fabrication Process

**Fig. 1:**
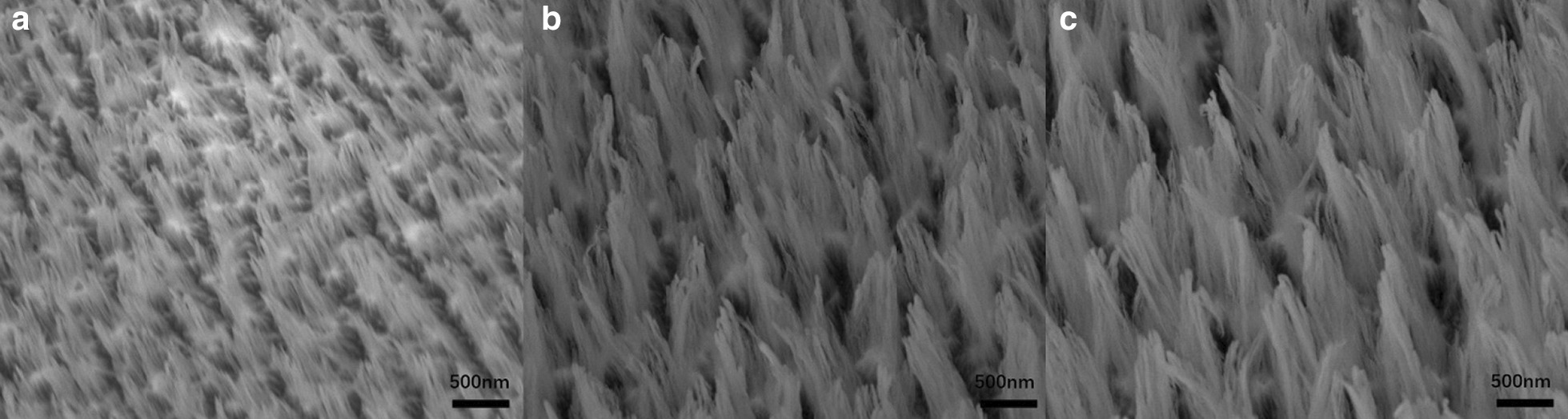
SEM images of GaAs substrate under different etching time

All samples were cut into 1.5 cm × 2 cm pieces of bulk GaAs, and the samples were pre-cleaned with conventional solvent and rinsed in deionized (DI) water. Then, the experiments were performed in an Oxford System100 etching reaction chamber, and the gases employed in this study were BCl_3_, Cl_2_, Ar, N_2_ and O_2_. A 5-min-long oxygen clean procedure was performed between each run to remove any polymer from the reactor sidewalls, minimize contamination and preserve process repeatability. The samples were loaded into the reactor by mounting them on an SiO_2_ carrier wafer, and since the sample was etched at room temperature, silicone grease was unnecessary before etching process [26]. As part of the optimization of the etching parameters, different etching time for measuring the process outcome was employed, as shown in Fig. 1.

## Characterization

The morphology analysis was characterized by scanning electron microscopy (SEM, FEI NanoSEM650, Hillsboro, OR, USA). The hydrophobic performance of the products was measured by a JC2000D water contact angle tester (Zhongchen digital technic apparatus co., ltd, Shanghai, China). The reflectivity of the sample was measured by a Agilent Cary7000 spectrophotometer.

## Results and Discussion

Figure 1 shows SEM images of GaAs substrate under different etching time. From the picture we can see that etching depth increases with the increment of etching time, but the morphology of the sample does not change greatly. After etching, the surfaces of GaAs samples become flocculent, relatively uniform in height but scattered around. When the oxygen flow is fixed and etching time is 3 min, the height of the etched sample is about 0.97–1.15 μm. As the etching time increases, the height of the formed structure will also increase. The height is 1.48–1.56 μm and 1.65–1.86 μm corresponding to the etching time of 4 min, 5 min. As the etched sample surface is divergent and scattered around, it is difficult to get an accurate value for pitch and period. This kind of flocculent structure greatly increases the specific surface area of the device and can be applied in the fields of supercapacitors and sensors.

**Fig. 2:**
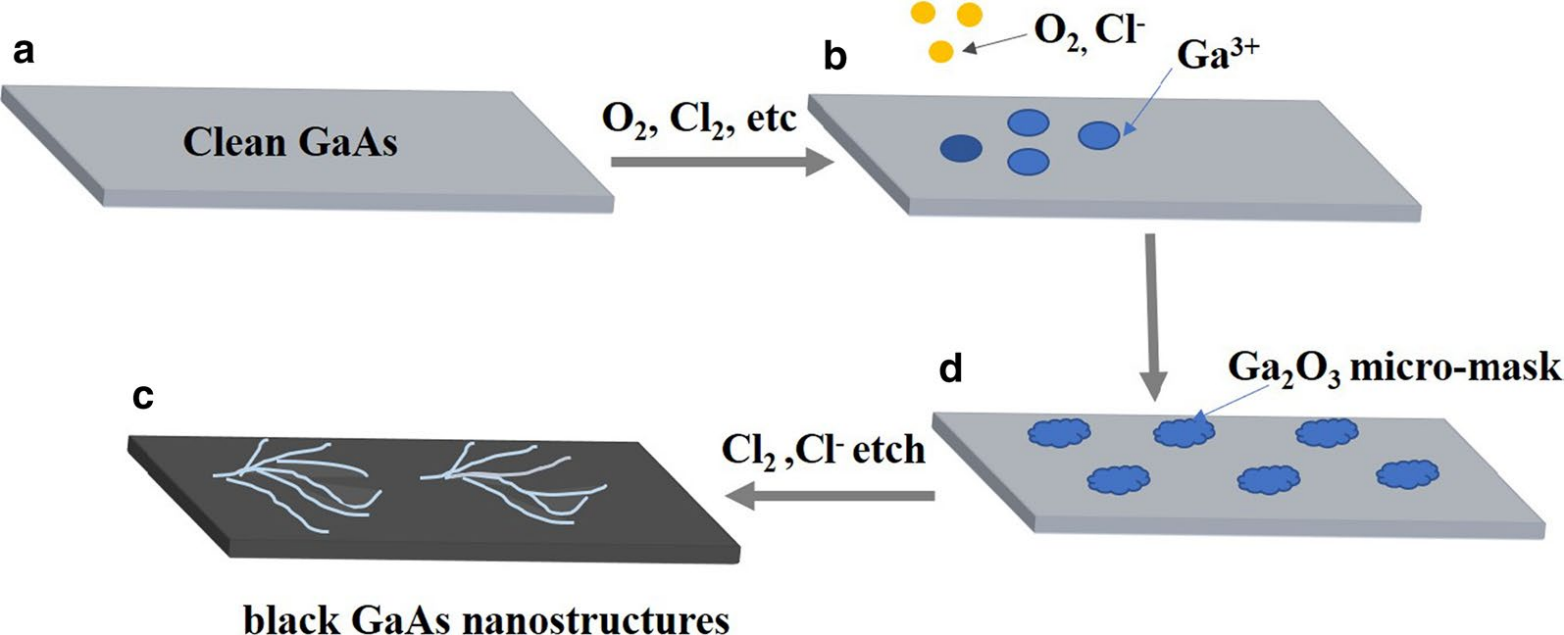
Schematic view of black GaAs nanostructure formation in plasma. **a** Clean GaAs; **b** random etching of the native oxide; **c** forming micro-mask; **d** forming black GaAs nanostructures

The etching mechanism of black GaAs is similar to that of black silicon. Under certain vacuum conditions, the etching gas is generated into plasma by glow discharge, which produces a large number of molecular free groups. Charged particles bombard the surface of the sample under the action of high-frequency electric field, while at the same time they react with some particles on the surface of GaAs, generating some volatile gas. The etching of GaAs surface is implemented under the dual role of physical bombardment and chemical reaction [27]. The entire etching process can be represented by following Fig. 2. Firstly, random etching of the native oxide (ions and oxygen) roughens the surface because of the forming of micro-mask [26, 28]. Then the lateral etching of microstructures on the substrate surface is inhibited by controlling the composition of etching gas and using the passivation of some products during etching [26], and the nanostructures on the substrate surface are obtained, namely the final black GaAs surface, as shown in Fig. 2d. All are done automatically in a single mask-less ICP process [27, 28].

**Fig. 3:**
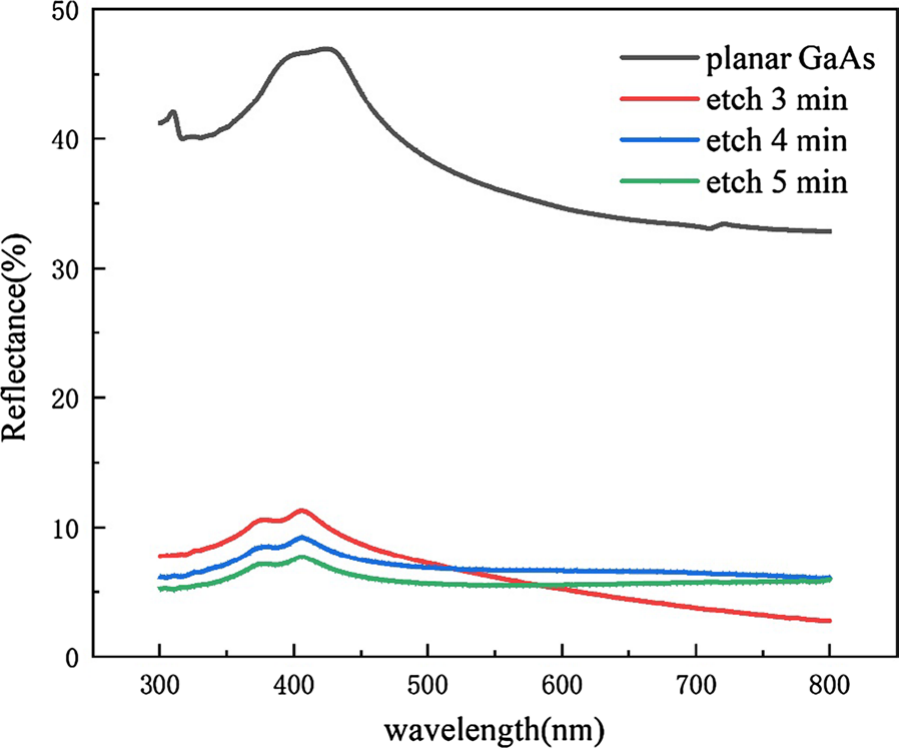
Reflectance of GaAs substrate under different etching time

**Fig. 4:**
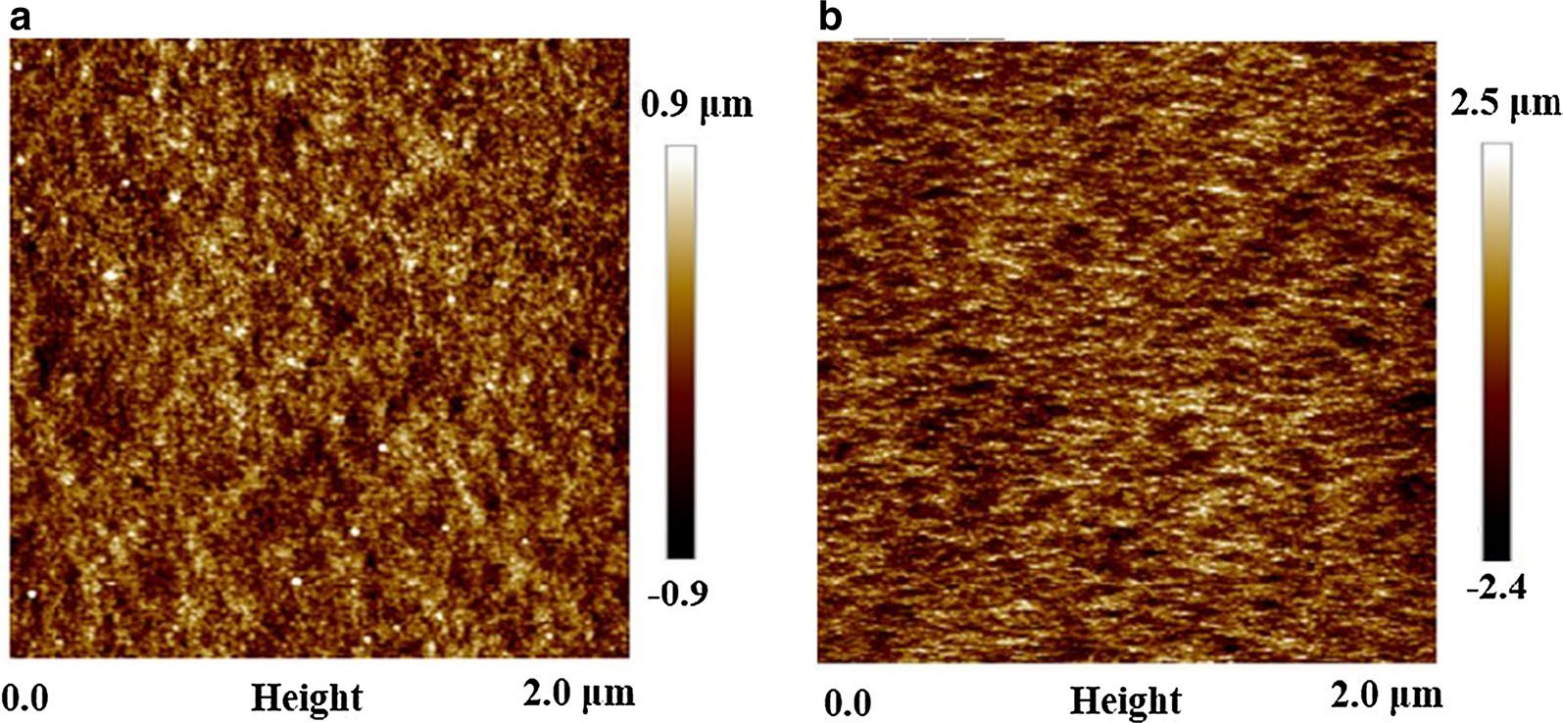
AFM images of **a** unetched GaAs sample; **b** black GaAs

We also tested the reflectivity of the prepared structure with Agilent's Cary 7000 spectrophotometer and found that the flocculent structure of GaAs sample had a very low reflectivity, as shown in Fig. 3. In the wavelength range of 590–800 nm, the reflectivity is 3 min < 5 min < 4 min. In the wavelength range of 400–590 nm, the reflectivity is 5 min < 4 min < 3 min. In the meantime, we can see that the reflectivity of the samples under different etching time is very low, with a difference of less than 1%. Considering the time and cost in the actual process, we choose 3 min as the fixed etching time in the subsequent experiments. We attribute the decrement of reflectivity to the rough structure formed on the GaAs surface. The sample formed a cluster structure after etching, and the roughened surface will limit the reflection of light and reduce the scattering of light, thus reducing the reflectivity of light. To verify our conclusion, AFM images were performed on the surface of the etched sample and the unetched sample, as shown in Fig. 4. The results show that the surface roughness of the etched sample is much larger than that of the unetched sample.

**Fig. 5:**
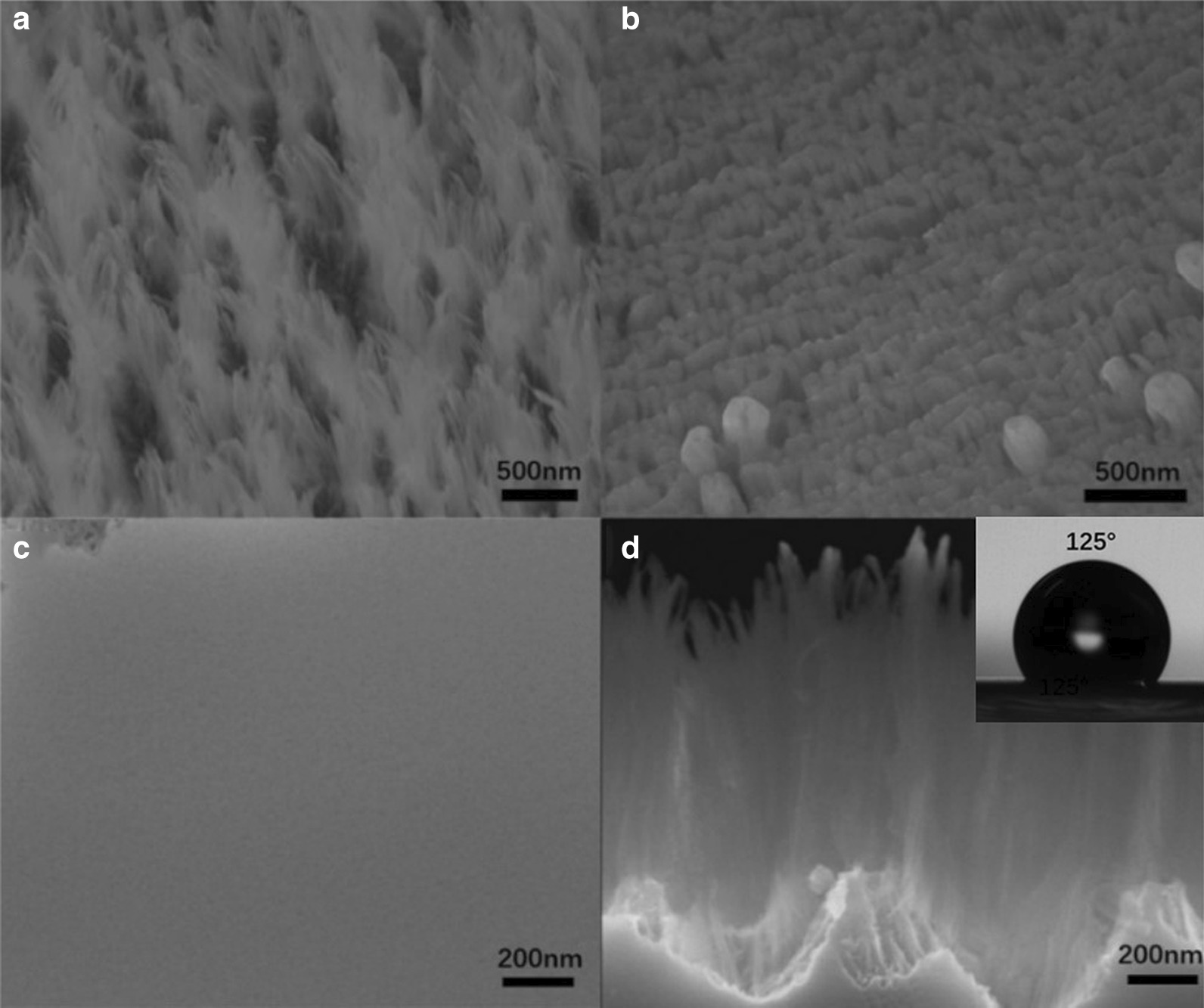
**a**–**c** SEM images of GaAs substrate under different oxygen flow rate; **d** the cross-sectional SEM image of GaAs substrate under the oxygen flow rate of 2

**Fig. 6:**
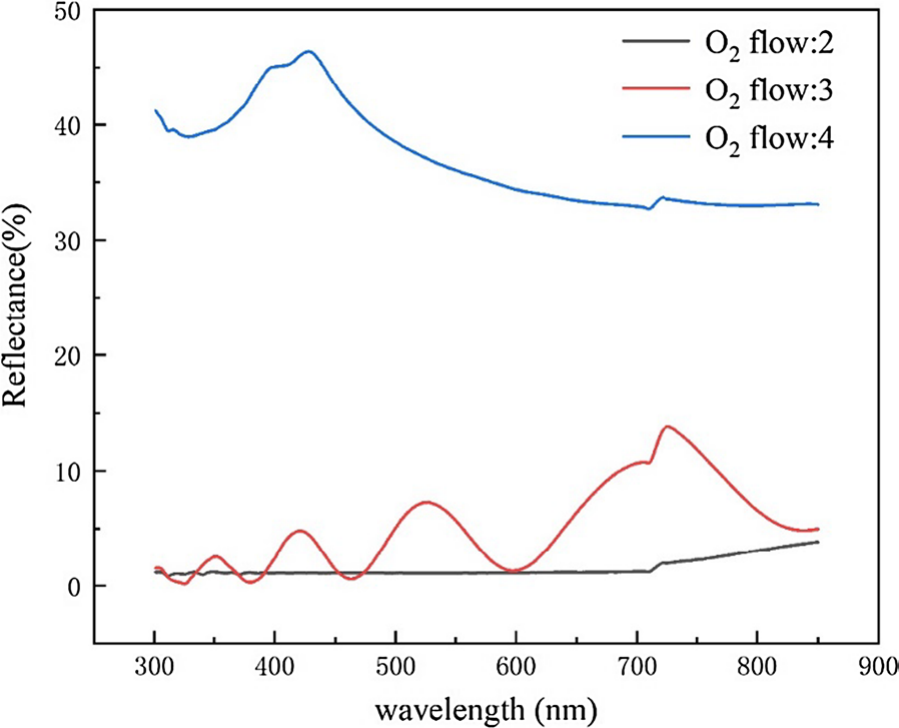
Reflectance of GaAs substrate under different oxygen flow rate

Then we investigate the effect of etching gas flow rate on the surface morphology and reflectivity of the sample when etching time is fixed at 3 min, and the oxygen flow was controlled. Here the role of oxygen is to form oxides during the etching process, and because of the different volatilization temperature during etching process, oxygen reacts with base atoms to form a micro-mask, thus affecting the etching result. Here, the oxygen flow ratio is set as 2:3:4, and the SEM images after etching are shown in Fig. 5. It can be seen from the figure that when the oxygen flow ratio is 3, the etched GaAs surface presents a neat columnar shape, and the height is 117–135 nm. When the oxygen flow ratio increases to 4, the GaAs surface becomes bright, and the sample surface is found to be smooth and without any pattern, as shown in Fig. 5c. The reason is that with the increment of oxygen, the proportion of Cl_2_ decreases, leading to the decrement of etching rate. The Ga ion in GaAs reacts with oxygen forming Ga_2_O_3_ as the micro-mask in the following etching process. However, excessive micro-mask will reduce the selective ratio of etching, resulting in the failure to form the black GaAs structure. That is why we see when the oxygen flow increased to 4 or more, the surface of the sample no longer appears black, but presents smooth and flat. Cary 7000 spectrophotometer was used to test the reflectivity of the three samples with different morphometry, and we found that the reflectivity increased gradually with the increase of oxygen flow. Figure 6 displays the reflectance of GaAs substrate under different oxygen flow rate. We can see that when the oxygen flow ratio is 2, the reflectivity has the lowest reflectivity, nearly to zero within the GaAs absorption range. The result is better than other nanostructures reported in the literature, such as nanowire, nanorod [29, 30]. This is because the flocculent surface of black GaAs greatly increased the propagation path of photons and reduced the reflection of light, while the etched sample with smooth surface presented high reflectivity. The structured GaAs sample also presented hydrophobicity with the contact angle of 125°, as shown in the enlarged SEM images of Fig. 5d, broadening the application range of black GaAs.

## Conclusions

In summary, we demonstrated a lithography-free ICP etching process for structuring GaAs surfaces with near-zero reflection (black GaAs). The structured sample displayed superior antireflective properties, yielding reflectance values as low as 0.093. The microstructures were obtained by only one-step ICP etching process and can be prepared in large scales. Moreover, the black GaAs sample presented hydrophobic property as the contact angle is 125°. This kind of structure is anticipated to absorb photon efficiently and reduce photon loss associated with light emission during charge recombination. The related preparation process also provides more possibilities for the preparation and development of GaAs devices.

## Data Availability

All data generated or analyzed during this study are included in this published article.

## Abbreviations

ICP: Inductively coupled plasma
CA: Contact angle
LEDs: Light-emitting diodes
PV: Photovoltaics
FETs: Field effect transistors
FDTD: Finite-difference time-domain
MacEtch: Metal-assisted chemical etching
DI: Deionized
SEM: Scanning electron microscopy
AFM: Atomic force microscopy
